# The role of working memory for task-order coordination in dual-task situations

**DOI:** 10.1007/s00426-021-01517-2

**Published:** 2021-04-21

**Authors:** Sebastian Kübler, Tilo Strobach, Torsten Schubert

**Affiliations:** 1 Department of Psychology, Martin-Luther University Halle-Wittenberg, Halle, Germany; 2 Department of Psychology, Humboldt-Universität Zu Berlin, Berlin, Germany; 3 Medical School Hamburg, Hamburg, Germany

## Abstract

Dual-task (DT) situations require task-order coordination processes that schedule the processing of two temporally overlapping tasks. Theories on task-order coordination suggest that these processes rely on order representations that are actively maintained and processed in working memory (WM). Preliminary evidence for this assumption stems from DT situations with variable task order, where repeating task order relative to the preceding trials results in improved performance compared to changing task order, indicating the processing of task-order information in WM between two succeeding trials. We directly tested this assumption by varying WM load during a DT with variable task order. In Experiment 1, WM load was manipulated by varying the number of stimulus–response mappings of the component tasks. In Experiment 2A, WM load was increased by embedding an additional WM updating task in the applied DT. In both experiments, the performance benefit for trials with repeated relative to trials with changed task order was reduced under high compared to low WM load. These results confirm our assumption that the processing of the task-order information relies on WM resources. In Experiment 2B, we tested whether the results of Experiment 2A can be attributed to introducing an additional task per se rather than to increased WM load by introducing an additional task with a low WM load. Importantly, in this experiment, the processing of order information was not affected. In sum, the results of the three experiments indicate that task-order coordination relies on order information which is maintained in an accessible state in WM during DT processing.

## Introduction

When performing two tasks simultaneously, performance decrements arise compared to situations in which the same tasks are performed in isolation (Koch et al., 2018). These dual-task (DT) costs are often explained by the limited attention capacity of the cognitive system resulting in a bottleneck during the processing of two temporally overlapping tasks (Pashler, 1994; Pashler & Johnston, 1989; Welford, 1952). According to the central bottleneck account, response selection for both tasks is executed sequentially. Therefore, during bottleneck processing of the first task the processing of the second task is interrupted and only continues after response selection for the first task has been completed. Over the last decades, it has been discussed whether this bottleneck constitutes a structural (McCann & Johnston, 1992; Pashler, 1994) or strategic (Fischer & Plessow, 2015; Meyer & Kieras, 1997) limitation of the cognitive system and whether resource allocation to the two tasks can take place in a more gradual and flexible rather than all-or-non fashion (Navon & Miller, 2002; Tombu & Jolicoeur, 2003). However, the question of how the processing order of the tasks is regulated at the bottleneck stage has been mostly neglected.

Different ideas have been proposed about the mechanisms which regulate the processing order in DTs. Earlier studies focused on the idea that task order is passively regulated by the central arrival times of the target stimuli. In that research vein it had been a decisive issue which of two task processing streams finishes perceptual processing first, and thus, reaches the bottleneck before to the other task. Accordingly, this difference in arrival times at the bottleneck determines the processing order in a rather first-come-first-serve principle (De Jong, 1995; Leonhard et al., 2011; Sigman & Dehaene, 2006; Strobach et al., 2018).

In addition to these earlier accounts, behavioral (Kübler et al., 2018; Luria & Meiran, 2003, 2006) and neuronal evidence (Schubert & Szameitat, 2003; Stelzel et al., 2008; Töllner et al., 2012) shows that task order is also coordinated top-down by executive control processes that actively schedule the processing of the component tasks in DT situations. It has been argued that these task-order coordination processes operate on an order control structure that contains information about the processing sequence of tasks and organizes the particular scheduling of the two task streams in each trial (De Jong, 1995; Luria & Meiran, 2003, 2006; see also Hirsch et al., 2017). While the role of this order control structure is well established (e.g. Kübler et al., 2019; Strobach et al., 2015), the locus of its processing is still a matter of debate. Some authors assume that the task-order control structure is actively maintained and processed in Working Memory (WM) during DT situations (Luria & Meiran, 2003, 2006). Direct evidence for this assumption, however, is still lacking. Thus, the aim of the current study was to elucidate the role of WM for task-order coordination in DT situations. In particular, we ask whether the maintenance and processing of the order control structure are subject to active WM-related processing or whether it is rather subject to a priming-related activation of memory traces from long-term memory. In addition to processing the order control structure, DT situations often require the monitoring of the stimulus sequence. This monitoring is necessary in many DT situations because participants are usually instructed to process the two tasks according to the order of stimulus presentation (Kübler et al., 2018; Strobach et al., 2018). As an additional question, we will also investigate whether these monitoring-related processes also rely on WM resources.

## Task-order coordination in DT situations

Evidence for the occurrence of task-order coordination processes stems from DT situations with variable order of the component tasks (De Jong, 1995; Kübler et al., 2018). For example, in a study by Szameitat et al. (2006; see also Luria & Meiran, 2003, 2006) the authors administered a DT consisting of an auditory (AUD) and a visual (VIS) choice RT task. The target stimuli of both tasks were presented in quick succession separated by a temporal interval of 200 ms. Furthermore, the sequence of stimulus presentation varied randomly from trial to trial such that either the auditory stimulus was presented first and the visual stimulus second (AUD–VIS trials) or the other way around (VIS–AUD trials). Importantly, participants were instructed to respond to both tasks according to the order of stimulus presentation. As a result, in each DT trial, participants had to monitor the sequence of stimuli and adjust their processing order accordingly imposing the requirement for task-order coordination processes.

For this DT situation, the authors distinguished two types of trials. In same-order trials, the order of both tasks in the current trial n was identical to the order of tasks in the preceding trial *n* – 1 (e.g., an AUD–VIS trial is preceded by an AUD–VIS trial). In different-order trials, in contrast, the task order in the current trial n was reversed relative to the previous trial n–1 (e.g., an AUD–VIS trial is preceded by a VIS–AUD trial). When comparing performance between these two trial types, RTs for both tasks were faster in same-order trials RTs in different-order trials. According to the authors (see also Kübler et al., 2019; Strobach et al., 2019), this performance benefit for same-order trials indicates the occurrence of task-order coordination processes, which rely on the active processing of task-order information in WM (De Jong, 1995; Luria & Meiran, 2003, 2006; Schubert, 2008; for a similar account on task-pair representations, see also Hirsch et al., 2017).

In more detail, Luria and Meiran (2003, 2006) suggested that task order in DT situations is regulated by a higher-order control structure, the task-order set. This task-order set contains information about the processing order of the component tasks and is activated in WM during the processing of a DT trial in addition to the task sets of the component tasks. Here, it guides the order of task processing by sequentially activating the task sets of the component tasks. After its implementation in WM, the task-order set remains active and, thus, affects performance in subsequent trials (see also Hirsch et al., 2017, 2018). In same-order trials, participants can apply the identical task-order set as in the preceding trial. This, in turn, results in faster RTs in same-order trials in comparison to different-order trials. In these different-order trials, a new task-order set has to be instantiated because the task-order set of the preceding trial does not specify the correct order in the current trial. Instantiating a new task-order set is more demanding and takes more time than re-applying the task-order set of the previous trial. This is so because this new task-order set is less activated compared with the task-order set of the previous trial. As a result, RTs in different-order trials are increased compared to RTs in same-order trials. This explanation for RT benefits in same-order compared with different-order trials due to active processing of the task-order set in WM is plausible and also in line with recent accounts on WM and its role for single as well as DT processing (Brass et al., 2017; Ellenbogen & Meiran, 2008; Oberauer et al., 2013; Schubert & Strobach, 2018). Importantly, as WM has only limited capacity, (Baddeley, 2003; Cowan, 2010), this explanation conceptualizes task-order coordination as a resource-dependent process.

Alternatively, rather than active processing in WM, the observation of performance benefits for same-order relative to different-order trials could reflect merely a consequence of automatic priming processes in long-term memory (Logan, 1988, 2002; Schneider & Logan, 2005; see also Hommel & Eglau, 2002; Mayr & Bryck, 2005; Waszak et al., 2003). For example, according to Logan’s instance theory (Logan, 1988, 2002; see also Hommel, 1998, 2004), during task processing task features, such as the order of the processed stimuli or of the processed motor response, are automatically encoded and stored as an integrated episodic trace in long-term memory. Future events that share features with the stored memory trace can cause its automatic retrieval. This retrieval of memory traces from prior task experience can then facilitate current task performance. Thus, in the context of task scheduling, repeating the task order of the previous trial may activate task-order information in long-term memory, which then would result in the performance benefits for same-order relative to different-order trials. Importantly, as this explanation relies on rather automatic priming processes in long-term memory that do not rely on the usage of WM resources, task-order coordination should not rely on the availability of WM resources.

## Rationale of the current study

**Fig. 1:**
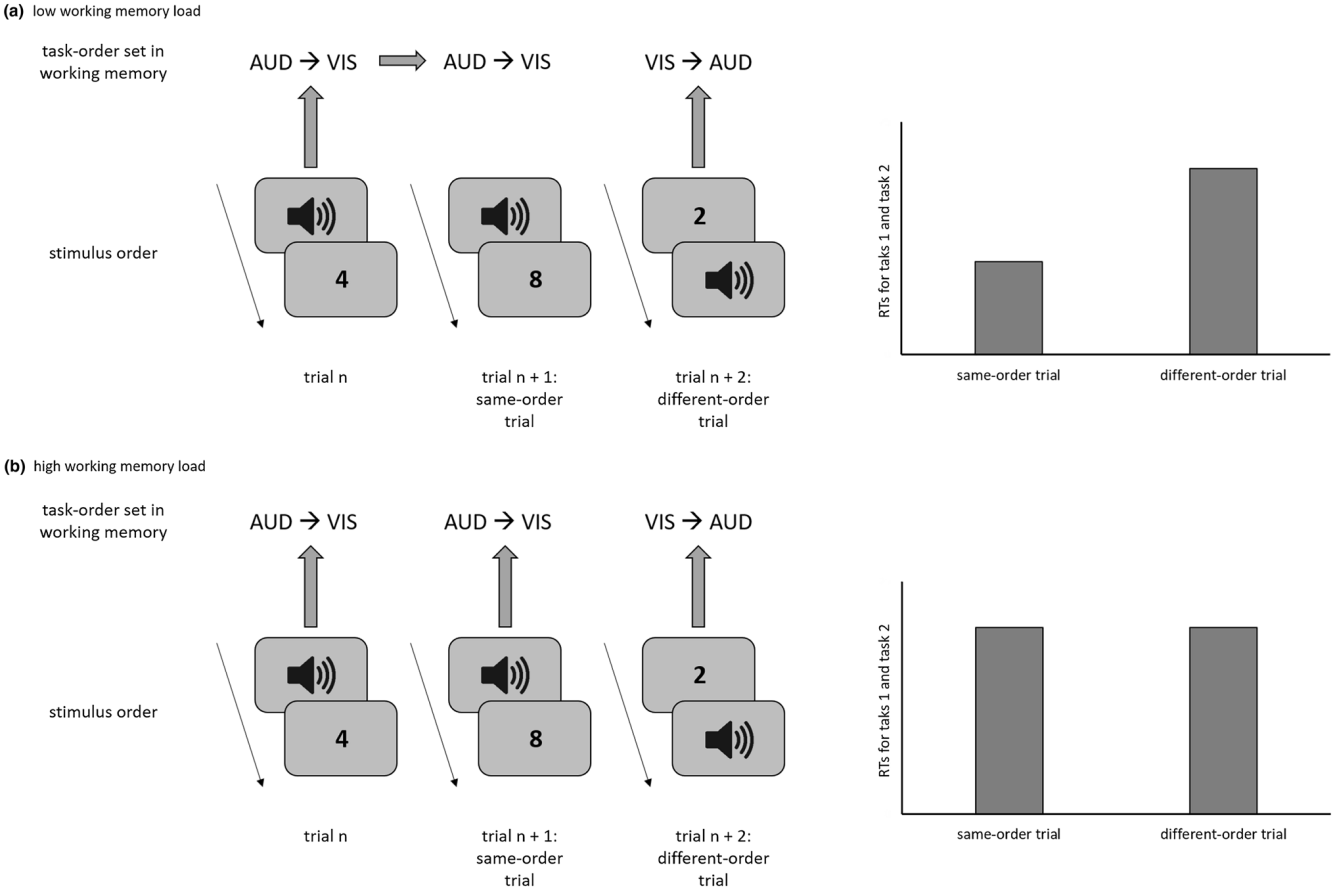
Left side: Exemplary sequence of three trials from random-order blocks including a same-order trial (trial *n* + 1) as well as a different-order trial (trial *n* + 2). Right side: Hypothesized results based on the assumption that the task-order set resides in working memory. **a** Low working memory load condition: Under the condition of sufficient working memory resources the task-order set can be maintained active in working memory throughout the entire course of a dual-task trial affecting performance in subsequent trials. In same-order trials, the task-order set of the preceding trial can be re-applied since stimulus order in the current trial is the same as in the previous trial. In different-order trials, on the other hand, a new task-order set has to be implemented in working memory because the task-order set of the previous trial does not match the stimulus order of the current trial. This results in a performance benefit for same-order compared with different-order trials reflected in faster RTs for task 1 and task 2. **b** high working memory load condition: Increasing working memory load hampers the maintenance and processing of the task-order set in working memory. In more detail, working memory resources do not suffice to keep the task-order set in an active state. As a result, in same-order trials participants cannot capitalize on the task-order set of the preceding trial and, instead, have to implement a new task-order set in working memory. Consequently, the performance benefit in same-order relative to different-order trials should be reduced (or even abolished). *AUD* auditory component task, *VIS* visual component task, *RT* reaction time

The main goal of the current study was to test, whether task-order coordination relies on the processing and maintenance of a task-order set in WM. To this end, in a series of experiments, we manipulated WM demands during a DT situation with variable task order. The rationale behind this manipulation is the following: WM is characterized by limited capacity (Baddeley, 2003; Cowan, 2010; and other authors). As an example, according to the model of Oberauer (2009, 2010), during task processing, relevant task representations have to be uploaded into an active and accessible state in WM to gain control over cognitive operations or actions. However, the amount of information that can be maintained in this state is limited. As a result, overload due to increasing WM demands during a DT situation should hamper the storage of and access to relevant task information. Applied to the current DT situation, we should find, increased RTs for both tasks in a high-load compared with a low-load DT situation. Most importantly, we also expect order-specific effects of the WM manipulation. If processing the task-order set indeed relies on WM resources during DT situations, then increasing WM demands should specifically hamper the maintenance and processing of the task-order set. As a result, in same-order trials, participants should not be able to make use of the task-order set from the preceding trial because the available WM resources do not suffice to keep the task-order set in an active state. Consequently, the performance benefit, i.e. faster RTs for both tasks, in same-order relative to different-order trials should be reduced (or even abolished) in a random-order DT situation with high WM load in comparison to a random-order DT situation with low WM load (Fig. 1). If, however, this performance benefit for same-order trials can be attributed to automatic priming by stored memory traces in long-term memory rather than to active processing in WM, then increasing WM load should not impair task-order processing in DT situations. Consequently, we should not find an effect of WM load on RT differences between same-order trials and different-order trials.

In addition to processing the task-order set in WM, a further demand in DTs with variable task order deals with monitoring related processes: In DT paradigms, participants are usually instructed to respond to both tasks according to the order of the stimuli. This does not only require participants to process and instantiate the order set in WM but also to monitor the stimulus sequence (Schubert & Szameitat, 2003; Stelzel et al., 2008). Evidence for this assumption stems from studies comparing DT performance from random-order blocks (i.e., blocks with randomly changing task orders) and conventional fixed-order blocks (i.e., blocks with constant, non-changing task order). Usually, in random-order blocks RTs for both tasks are increased compared to fixed-order blocks. These performance differences have often been attributed to different demands on monitoring-related processes in both block types. In fixed-order blocks participants know the order of tasks in advance and task order does not vary throughout the block. As a result, participants can employ a constant scheduling strategy and do not need to monitor the order of stimuli. In random-order blocks, due to instruction, participants have to match their processing order to the variable stimulus order. As a result, in each trial, they have to monitor the sequence of stimulus presentation. These differences in demands on monitoring-related processes are often used to explain performance differences between fixed-order and random-order blocks. While these two block types may also differ with respect to other cognitive processes beyond monitoring, such as divided attention or the requirement to switch task order, monitoring seems to be an important distinguishing feature between both block types.

Evidence for the importance of monitoring processes in random-order blocks stems, for example, from a study by Kübler et al. (2018). In this study, the authors reduced demands on monitoring in random-order blocks by allowing for free order choices. As a result, participants did not require to monitor the sequence of stimulus presentation but instead could base their processing order on their individual order choice. Importantly, when reducing demands on monitoring related processes, RTs in random-order blocks were significantly reduced almost to the level of performance in fixed-order blocks (see also Strobach et al., 2019 for a similar study). In sum, these results suggest that in addition to processing and changing a task-order set, a further and important demand in random-order blocks is the monitoring of the stimulus sequence.

Importantly, however, increasing WM load may also disturb these monitoring-related processes (instead or in addition to a potential effect on the processing and maintenance of the task order set). To test whether WM load has an effect on such monitoring processes, we administered fixed-order blocks in addition to the random-order blocks in the current experiment. In fixed-order blocks, the task order remains constant throughout a block and, as a consequence, there is no requirement for additional monitoring processes. Thus, we can compare performance, i.e. RTs for both tasks, between fixed-order blocks and random-order blocks, which provides us with an indicator for monitoring related processes (Stelzel et al., 2008; Szameitat et al., 2002). To test whether the applied WM manipulation affects monitoring related processes, we can then contrast this difference between fixed-order and random-order DT blocks between both load conditions. If monitoring is hampered by increased WM load, we should find increased RT differences for both tasks between random-order blocks and fixed-order blocks under high compared to low WM load. If monitoring does not rely on WM resources, the WM manipulation should not affect this RT difference between both block types.

We conducted a series of three experiments with different WM manipulations. In Experiment 1, we varied the size of the task sets of the component tasks to be held active in WM by manipulating the number of stimulus–response mappings. In Experiment 2A, we administered a WM updating task in addition to the DT situation to increase the overall WM load of the task situation. In Experiment 2B, we introduced an additional task with low demands on WM to test, whether the implementation of an additional task and the need to switch between these tasks or whether increased WM load can be attributed to the results of Experiment 2A.

## Experiment 1

In Experiment 1, we manipulated WM demands by varying the size of the task sets of the component tasks (Hick, 1952; Kikumoto & Mayr, 2017; Oberauer, 2009; Schubert & Strobach, 2018; Stelzel et al., 2008). In low-load blocks, participants had to maintain two stimulus–response mappings for each component task in WM, while in high-load blocks, they had to maintain four stimulus–response mappings for each task.

## Material and methods

### Participants

Twenty-four (21 female) right-handed participants aged from 18 to 30 (mean age 22) were recruited from a participant pool at the Institute of Psychology at the Humboldt-Universität in Berlin. Participants were informed about the experimental procedure and gave their consent to participate in the study in advance. As compensation, they received either course credit or 8 euros per hour. Data of one participant were excluded due to a high number of erroneous trials (only 55% correct trials).

### Stimuli and task

Participants were seated in front of a 24 inch LCD monitor with a 1920 × 1080 pixel resolution and a 144 Hz refresh rate at a viewing distance of 80 cm while performing a DT consisting of an auditory and a visual choice RT task (Stelzel et al., 2008). For the visual task, one of four digits (2, 4, 6, or 8) was presented centrally on a computer screen (0.52° × 0.31°). Responses on the visual stimuli were mapped on the ‘M’, ‘,’, ‘.’, and ‘-’ buttons of a QWERTZ keyboard in ascending order, and participants were instructed to respond using their right index, middle, ring or little finger, respectively. In the auditory task, participants responded to one of four tones with different pitches (150 Hz, 550 Hz, 950 Hz, or 1350 Hz) presented via headphones by pressing the ‘*Y*’, ‘*X*’, ‘*C*’, and ‘*V*’ buttons with their left little, ring, middle and index finger. Participants were instructed to respond to both stimuli as accurately and fast as possible and in the same order they were presented in.

### Design and procedure

Each DT trial started with a fixation mark that was presented for 750 ms followed by a blank screen for 250 ms. Subsequently, an auditory and a visual stimulus were presented for 200 ms each; stimulus onsets were separated by a time interval of 200 ms. Following the stimuli, the screen was cleared. After responses to both target stimuli or after expiration of a maximal response period of 2750 ms, the next trial began after an inter-trial interval (ITI) of 1250 ms. Error feedback for omitted responses as well as incorrect stimulus discrimination was presented centrally for 500 ms during the ITI and consisted of the German words ‘ZU LANGSAM’ (too slow) or ‘FALSCH’ (incorrect). The timing of single-task trials during the practice phase (see below) was similar with the difference that the response period started after the offset of the first stimulus and without the presentation of a second stimulus.

In total, participants performed twelve blocks with random task order. These blocks consisted of 33 trials each. In these random-order blocks, the order of stimuli varied so that half of the trials were AUD–VIS and the other half VIS–AUD trials. Importantly, the order of stimuli was unpredictable and half of the trials were same-order and the other half different-order trials. The sequence of these same-order and different-order trials was randomized within each block. In sum, this resulted in 16 same-order and 16 different-order trials for each block (the additional first trial of each block was removed from analyses as it neither constitutes a same-order nor a different-order trial).

WM load was manipulated by introducing blocks with different numbers of stimulus–response mappings (Stelzel et al., 2008). Throughout high-load blocks, all four visual and all four auditory stimuli were presented as target stimuli, which resulted in eight stimulus–response mappings participant had to maintain active in WM. In low-load blocks, on the contrary, only two stimuli of each task (the digits ‘4’, and ‘6’ for the visual as well as 550 Hz and 950 Hz tones for the auditory task) were presented, which yielded four stimulus–response mappings that had to be maintained in WM. For both, the visual and the auditory task, the intermediate stimuli (differing from each other by the same degree as in high-load blocks) were selected as target stimuli in low-load blocks to keep the difficulty of stimulus discrimination constant between both load conditions (Maquestiaux et al., 2008). Participants were informed about the load condition prior to each block. Half of the random-order blocks were low-load and the other half were high-load blocks, which resulted in six blocks for each condition. Combining the two factors task order (same-order trials, different-order trials) and WM load (low-load blocks, high-load blocks) in a 2 × 2 design resulted in four DT conditions: Same-order trials and different-order trials from low-load and high-load blocks, respectively. We compared the RT difference between same-order and different-order trials under low and high WM load. In addition to random-order blocks, we presented four fixed-order blocks with high and low WM load. This allowed us to test whether monitoring related processes, which are required in random-order but not in fixed-order blocks, are affected by increasing WM demands.

The experiment started with a practice phase. For each component task, participants performed two single-task blocks with 20 trials each. Half of the participants started with two auditory single-tasks blocks, the other half of the participants started with two visual single-task blocks. For each component task, participants were presented a single-task block with four and a single-task block with eight stimulus–response mappings in counterbalanced order. Then participants performed two random-order blocks à 20 trials for each load condition in counterbalanced order. In the main part of the experiment, participants first performed twelve random-order DT blocks with 33 trials each. Half of the participants first performed six of these random-order blocks under low load and then six blocks under high load. The other half of the participants performed these 12 blocks in the reversed order. After finishing random-order blocks, participants were presented four fixed-order blocks à 48 trials (for an identical sequence of block types, see Kübler et al., 2018), two for each possible task order (AUD–VIS, VIS–AUD). One group of participants first performed these blocks in the low-load condition and then in the high-load condition, whereas for the other group of participants this sequence was reversed. The order of AUD–VIS and VIS–AUD fixed-order blocks was counterbalanced across participants. In fixed-order blocks, the order of stimuli did not vary. As a result, this block type does not include any order changes but only one type of trial with a fixed order. Furthermore, participants were informed about the specific order individually for each block beforehand. As a result, they could employ a constant scheduling order and were not required to employ additional monitoring-related processes.

## Results

We analyzed mean RTs and error rates for both component tasks. For each participant, trials from practice blocks and the first trial of each random-order block were withdrawn from all analyses. For analyses of RTs, erroneous trials (discrimination errors and trials with incorrect task order, *mean*[*m*] = 18%), as well as trials with RTs slower and faster than ± 2.5 standard deviation from the mean of each factor combination (*m* = 2%), were removed from analyses for each participant. RTs and error rates were aggregated across AUD–VIS and VIS–AUD trials. In the first step, we only analyzed performance in random-order blocks. More specifically, we analyzed performance in same-order and different-order trials under low and high WM load using analyses of variance (ANOVAs) with the within-subjects factors WM LOAD (low-load blocks, high-load blocks) and TASK ORDER (same-order trials, different-order trials) separately for the first performed task—task 1—and the second performed—task 2. In the second step, we analyzed participants’ performance in fixed- and random-order block using an ANOVA with the within-subjects factors WM LOAD (low-load blocks, high-load blocks) and BLOCK TYPE (fixed-order blocks, random-order blocks). Please note, that fixed-order blocks cannot be subdivided further in different trial types (since all trials are presented with a constant stimulus order and task order does not vary). As a consequence, for this analysis we collapsed performance across same-order and different-order trials, i.e. both trial types from random-order blocks.

In addition, we report Bayesian analyses using JASP software (Wagenmakers et al., 2018) where conventional frequentist analyses may not be sufficient to draw clear conclusions. These analyses specify, for example, any lack of significance when testing performance differences between same-order and different-order trials. Furthermore, we applied a Bayesian repeated-measures ANOVA (van den Bergh et al., 2020) for the comparisons between fixed-order and random-order blocks. In particular, this was done to provide further evidence for or against the assumption that increasing WM load affects monitoring related processes in random-order blocks. For the purpose of this Bayes analysis, we calculated the posterior probabilities of a model not including the interaction of the factors WM LOAD and BLOCK TYPE and compared it to a model containing this interaction. Importantly, the Bayes factor BF_01_ provides information which of these two models better fit the data, with values larger than 1 providing evidence for the model not including the interaction effect and values smaller than 1 providing evidence for a model including this interaction (Raftery, 1995; Wagenmakers, 2007). A model not including the TASK × ORDER interaction would support the idea that increasing WM load would have no effect monitoring-related processes, whereas a model specifying this interaction would indicate the modulation of monitoring-related processes by WM load.

### Comparison between same-order and different-order trials

**Fig. 2:**
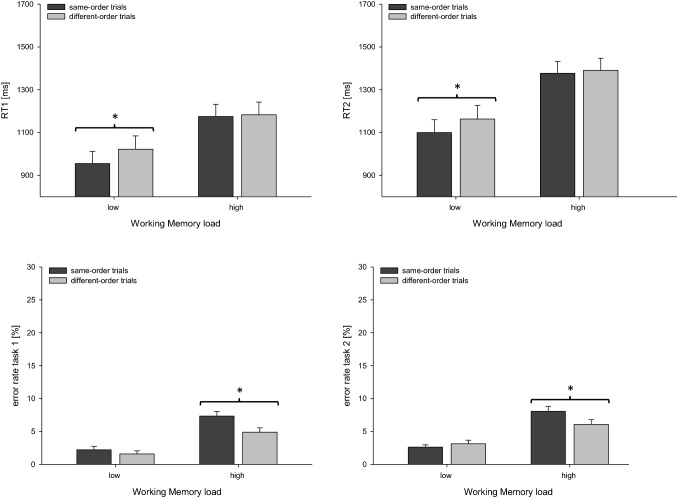
Mean RTs and error rates for task 1 and task 2 as a function of trial type and Working Memory load for Experiment 1. Error bars reflect the standard error of the mean. Asterisks indicate significant differences between same-order and different-order trials conditions. Left panels: reaction times (RT 1, upper panel) and error rates (lower panel) for task 1, right panels: reaction times (RT 2, upper panel) and error rates (lower panel) for task 2

As can be seen in Fig. 2, RTs for task 1 (RT 1) were significantly increased in high-load blocks (*m* = 1179 ms) compared to low-load blocks (*m* = 989 ms), *F*(1, 22) = 101.178, *p* < 0.001, *η*_*p*_^*2*^ = 0.821, indicating a general decrement in performance under high WM load. In line with previous research on task-order coordination (De Jong, 1995; Kübler et al., 2018), responses for task 1 were faster in same-order trials (*m* = 1065 ms) than those in different-order trials (*m* = 1103 ms), *F*(1, 22) = 15.307, *p* = 0.001, *η*_*p*_^*2*^ = 0.410.

Importantly, as indicated by the significant two-way interaction, *F*(1, 22) = 17.435, *p* < 0.001, *η*_*p*_^*2*^ = 0.442, this performance benefit for same-order compared to different-order trials was modulated by the factor WM LOAD. In low-load blocks, RT 1 was significantly shorter in same-order trials (*m* = 955 ms) than in different-order trials (m = 1022 ms), *t*(22) = 5.523, *p* < 0.001, *d* = 1.151. Contrarily, in high-load blocks, RT 1 in same-order (*m* = 1175 ms) and in different-order trials (*m* = 1183 ms) did not differ significantly, *t*(22) = 0.728, *p* = 0.475, *d* = 0.103 which was also supported by an *BF*_*01*_ = 3.600. Thus, in line with our assumption, increasing WM demands resulted in a reduced performance benefit for same-order versus different-order trials.

Analyzing accuracy in task 1, we observed more errors in high-load (*m* = 6.1%) relative to low-load blocks (*m* = 1.9%), *F*(1, 22) = 57.870, *p* < 0.001, *η*_*p*_^*2*^ = 0.725. Overall, mean error rates decreased from same-order trials (*m* = 4.8%) to different-order trials (*m* = 3.2%), *F*(1, 22) = 11.199, *p* = 0.003, *η*_*p*_^*2*^ = 0.337. Additionally, this decrease in errors from same-order to different-order trials was modulated by the factor WM LOAD, *F*(1, 22) = 5.832, *p* = 0.024, *η*_*p*_^*2*^ = 0.210. While under low load error rates in task 1 did not differ between same-order trials (*m* = 2.2%) and different-order trials (*m* = 1.6%), *t*(22) = 1.297, *p* = 0.208, *d* = 0.212 *BF*_*01*_ = 2.179, under high load, we found a performance benefit for different-order trials (*m* = 4.9%) compared to same-order trials (*m* = 7.3%), *t*(22) = 3.583, *p* = 0.002, *d* = 0.740. In sum, under low load we could not find any performance difference between same-order and different-order trials on the basis of task 1 errors, while under high load we found a benefit for different-order compared to same-order trials. This pattern of results for error rates might be explained by a possible speed-accuracy tradeoff. More specifically, while in the low-load condition participants can re-apply the task-order set of the previous trial in same-order trials, in the high-load condition demands on task-order coordination are increased in same-order trials since the task-order set of the previous trial does not reside in WM anymore and a new task-order set has to be activated. This might result in a more cautious processing strategy resulting in increased RTs and concurrently reduced error rates.

For RTs in task 2 (RT 2), a similar pattern of results was identified as compared to RT 1. We observed a reliable main effect of WM LOAD indicating shorter RT 2 in low-load blocks (*m* = 1131 ms) compared to high-load blocks (*m* = 1383 ms), *F*(1, 22) = 138.870, *p* < 0.001, *η*_*p*_^*2*^ = 0.863. Additionally, we found a significant effect of the factor TASK ORDER, *F*(1, 22) = 16.413, *p* = 0.001, *η*_*p*_^*2*^ = 0.427, reflecting a RT benefit for same-order trials (*m* = 1239 ms) relative to different-order trials (*m* = 1277 ms).

Similar to RT 1, we also found a significant interaction of these two factors, *F*(1, 22) = 7.731, *p* = 0.011, *η*_*p*_^*2*^ = 0.260. Further analyses revealed that in low-load blocks RT 2 was shorter in same-order trials (*m* = 1100 ms) compared to different-order trials (*m* = 1163 ms), *t*(22) = 5.364, *p* < 0.001, *d* = 1.127. In high-load blocks, the difference between same-order trials (*m* = 1377 ms) and different-order trials (*m* = 1390 ms) did not reach significance, *t*(22) = 0.984, *p* = 0.336, *d* = 0.071, *BF*_*01*_ = 2.964, mirroring the findings in task 1.

In task 2, participants conducted more errors when WM demands were increased in high-load blocks (*m* = 7.1%) compared to low-load blocks (*m* = 2.9%), *F*(1, 22) = 76.675, *p* < 0.001, *η*_*p*_^*2*^ = 0.777. The main effect of TASK ORDER did not reach significance, *F*(1, 22) = 2.005, *p* = 0.171, *η*_*p*_^*2*^ = 0.084. The interaction of both factors was significant *F*(1, 22) = 5.324, *p* = 031, *η*_*p*_^*2*^ = 0.195. The non-significant but numerical benefit on the level of task 2 errors for same-order (*m* = 2.6%) relative to different-order trials (*m* = 3.1%) under low WM load, *t*(22) = 0.885, *p* = 0.386, *d* = 0.186, *BF*_*01*_ = 3.260 was reversed in high-load blocks; participants conducted fewer errors in different-order (*m* = 6.1%) compared to same-order trials (*m* = 8.1%), *t*(22) = 2.197, *p* = 0.039, *d* = 0.452. Analogously to error rates in task 1, this finding might be explained by a potential speed-accuracy tradeoff (see also task 1 error rates). In sum, increasing WM load reduced performance benefits for same-order trials on the level of RT and error rates for task 1 and task 2. This is in line with the assumption that the task order set cannot be processed efficiently in WM under high load,

### Comparison between fixed-order and random-order blocks

In the next step, we compared RTs and error rates from fixed-order blocks and random-order blocks under both load conditions. This was done to test whether increasing WM demands may affect monitoring related processes necessary for DT with variable task order (Kübler et al., 2018). The corresponding ANOVA (all data for the block comparison can be found in Table 1) revealed that RT 1 was increased in high-load blocks (*m* = 1065 ms) compared to low-load blocks (*m* = 863 ms), *F*(1, 22) = 177.678, *p* < 0.001, *η*_*p*_^*2*^ = 0.890. In addition, we found a reliable effect of the factor BLOCK TYPE, *F*(1, 22) = 62.888, *p* < 0.001, *η*_*p*_^*2*^ = 0.741, mirrored in increased RT 1 in random-order blocks (*m* = 1084 ms) compared to fixed-order blocks (*m* = 845 ms) and indicating the occurrence of monitoring related processes. Importantly, this increase from fixed-order to random-order blocks did not differ between load conditions, as was indicated by the non-significant interaction of the two factors, *F*(1, 22) = 0.535, *p* = 0.472, *η*_*p*_^*2*^ = 0.024. This was also supported by a Bayes Factor of *BF*_*01*_ = 3.110 from the respective model comparison, providing evidence for a model only containing the main effects WM LOAD and BLOCK TYPE without further specifying an interaction of these two factors. Thus, we can conclude that increasing WM demands did not affect monitoring related processes.

**Table 1:** Mean reaction times (RTs) in ms (and standard deviation) and error rates in % for task 1 and task 2 in fixed-order and random-order blocks as a function of Working Memory (WM) load for Experiment 1 and Experiment 2A

	WM load			
	Low		High	
	Fixed-order block	Random-order block	Fixed-order block	Random-order block
Experiment 1				
RT 1	738 (204)	989 (282)	951 (211)	1179 (276)
RT 2	851 (230)	1131 (296)	1155 (226)	1384 (264)
error rate task 1	1.9 (2.7)	1.9 (2.1)	5.3 (3.4)	6.1 (2.8)
error rate task 2	3.2 (3.1)	2.9 (1.7)	7.4 (4.2)	7.1 (2.8)
Experiment 2A				
RT 1	885 (325)	1215 (409)	1047 (355)	1319 (438)
RT 2	1031 (323)	1372 (407)	1215 (378)	1498 (465)
error rate task 1	1.0 (1.5)	2.4 (1.6)	2.4 (2.2)	4.0 (2.8)
error rate task 2	3.5 (3.1)	4.6 (2.9)	4.5 (3.7)	5.1 (3.2)

When analyzing the error data in task 1, only the factor WM LOAD modulated the frequency of incorrect responses in task 1, *F*(1, 22) = 70.882, *p* < 0.001, *η*_*p*_^*2*^ = 0.763, with more errors being committed in high-load (*m* = 5.7%) compared to low-load (*m* = 1.9%) blocks. Neither the effect of the factor BLOCK TYPE, *F*(1, 22) = 0.457, *p* = 0.506, *η*_*p*_^*2*^ = 0.020, nor the interactions of the two factors, *F*(1, 22) = 2.124, *p* = 0.159, *η*_*p*_^*2*^ = 0.088, was significant. The non-significant interaction is also supported by a Bayes factor of *BF*_*01*_ = 2.561, providing positive evidence for a model that does not specify the interaction of the two factors.

Also for task 2, increased WM demands in high-load blocks resulted in longer RTs (*m* = 1269 ms) relative to low-load blocks (*m* = 991 ms), *F*(1, 22) = 316.433, *p* < 0.001, *η*_*p*_^*2*^ = 0.935. Also, the factor BLOCK TYPE reached significance, *F*(1, 22) = 62.886, *p* < 0.001, *η*_*p*_^*2*^ = 0.741, with increased RT 2 in random-order (*m* = 1258 ms) relative to fixed-order blocks (*m* = 1003 ms). Similarly to RT 1, this effect of BLOCK TYPE did not differ between both load conditions, as was confirmed by the non-significant interaction of WM LOAD and BLOCK TYPE, *F*(1, 22) = 2.629, *p* = 0.119, *η*_*p*_^*2*^ = 0.107. Similarly, the respective model comparison yielded a Bayes factor of *BF*_*01*_ = 2.213, providing no evidence for a modulation of performance differences between both block types due to WM load (i.e., the model does not benefit from the additional inclusion of the interaction of WM LOAD and BLOCK TYPE).

Participants produced more task 2 errors in high-load (*m* = 7.3%) compared to low-load blocks (*m* = 3.1%), *F*(1, 22) = 67.832, *p* < 0.001, *η*_*p*_^*2*^ = 0.755. The effect of the factor BLOCK TYPE, *F*(1, 22) = 0.517, *p* = 0.480, *η*_*p*_^*2*^ = 0.023, and the interactions of the two factors, *F*(1, 22) = 0.022, *p* = 0.884, *η*_*p*_^*2*^ = 0.001, did not reach significance levels. Further support for the non-significant interaction comes from the Bayesian model comparison with a Bayes Factor of *BF*_*01*_ = 3.320. In sum, these results suggest that increasing WM demands in high-load blocks did not affect monitoring related processes. This was indicated by no differences in performance decrements in random-order compared with fixed-order blocks between both load conditions.

However, the observed performance differences between fixed-order and random-order blocks might also be explained due to practice over the course of the experiment rather than different demands on task-order coordination. This is especially important since participants showed better performance in fixed-order blocks which were presented after random-order blocks. To exclude that the performance differences between both block types can be exclusively explained by practice effects, we compared RTs in the last random-order block with RTs in the (succeeding) first fixed-order blocks. Please note, that the assumption of potential training effects predicts a continuous improvement with subtle changes from block to block throughout the entire experiment rather than a sudden improvement in performance between two succeeding blocks. Importantly, when comparing the last random-order block with the first fixed-order block, we found a rapid improvement in performance. RT 1 was significantly slower in the last random-order block (*m* = 1053 ms) compared with the first fixed-order block (*m* = 809 ms), *F*(1, 22) = 16.384, *p* = 0.001, *η*_*p*_^*2*^ = 0.427. Also, RT 2 abruptly decreased from the last random order block (*m* = 1211 ms) to the first fixed-order block (*m* = 983 ms), *F*(1, 22) = 10.131, *p* = 0.004, *η*_*p*_^*2*^ = 0.315. Please note, that this RT difference between the last random-order and the first fixed-order block is similar compared to contrasting random-order and fixed-order blocks collapsed over the entire experiment. Furthermore, in our view, such rapid changes in performance cannot exclusively be accounted for by practice effects. Rather, they are in line with the assumption that both fixed-order and random-order blocks differ in the requirement to employ task-order coordination processes. In the next step, we compared performance in the first random-order block with performance in the last fixed-order block. Also this comparison revealed that RT 1 was slowed down in random-order (*m* = 1121 ms) compared with fixed-order blocks (*m* = 876 ms), *F*(1, 22) = 11.678, *p* = 0.002, *η*_*p*_^*2*^ = 0.347. Similarly, for RT2 a similar pattern was observed with slower RT in random-order (*m* = 1304 ms) compared with fixed-order blocks (*m* = 1005 ms), *F*(1, 22) = 14.683, *p* = 0.001, *η*_*p*_^*2*^ = 0.400. Importantly, RT differences between the last random-order block and the first fixed-order block (task 1: *m* = 244 ms; task 2: *m* = 228 ms) did not differ compared with RT differences between the first random-order block and the last fixed-order block (task 1: *m* = 228 ms; task 2: *m* = 299 ms), *t*(22) = 0.006, *p* = 0.996, *d* = 0.001 for task 1 and *t*(22) = 0.556, *p* = 0.584, *d* = 0.058 for task 2. Additional Bayesian *t*-tests supported these results with *BF*_*01*_ = 4.573 for task 1 and *BF*_*01*_ = 3.974 for task 2, providing no evidence for any differences between fixed-order and random-order blocks across the course of the experiment. These findings indicate that the difference between fixed-order and random-order blocks can for the most part be accounted for by increased demands on task-order coordination. Thus, based on the additional analyses, we conclude that a performance difference between fixed-order and random-order blocks cannot entirely be explained by the potential practice effect.

## Discussion

In Experiment 1, manipulating WM load modulated the performance benefits for same-order relative to different-order trials. In particular, under low load, performance was facilitated in same-order compared with different-order trials replicating earlier findings (Kübler et al., 2018; Luria & Meiran, 2003). In high-load blocks, increasing the number of stimulus–response mappings resulted in a reduction of these performance benefits in task 1 and task 2. These results are in line with the assumption that the processing of the task-order set relies on WM resources and that increasing WM load hampers the processing and maintenance of a task-order set. Furthermore, the current findings are not consistent with the assumption that the performance benefit for same-order trials is due to a rather automatic priming in long-term memory (Logan, 1988; see also Hommel, 1998; Hommel, 2004). If this was the case, we should have found that WM load does not affect the performance benefits for same-order trials.

Additionally, we did not find evidence for the assumption that increasing WM load affects monitoring related processes that are necessary to adjust the task order to the order of stimulus presentation (Kübler et al., 2018; Stelzel et al., 2008). In this case, we should have found larger RT increases from fixed-order to random-order blocks under the high compared to the low-load condition. Instead, we did not find evidence for a modulatory effect of WM load on the performance differences between fixed-order and random-order which was also confirmed by a Bayesian model comparison. Thus, these findings do not support the assumption that the monitoring of the stimulus sequence in DTs relies on available WM resources.

While these findings are suggestive for the assumption that the task-order set is maintained and processed in WM during DT processing, an important methodological confound needs to be resolved before we can assess the reliability of this conclusion. In more detail, the findings of Experiment 1 could also be explained by a different number of stimulus and response repetitions between high- and low-load conditions. In more detail, we presented two stimuli with two responses and four stimuli with four responses in low-load and high-load blocks, respectively. As a result, there was a higher frequency of stimulus and response repetitions for both tasks in same-order trials of low-load blocks (0.5 × 0.5 = 0.25) compared to same-order trials of high-load blocks (0.25 × 0.25 ≈ 0.06). Irrespective of the actual task order, however, repeating stimulus and response features on two succeeding trials may result in performance facilitation (Hommel et al., 2004; Mayr et al., 2003). Thus, increased numbers of stimulus and response repetitions in low compared to high-load blocks could also explain the results we found in Experiment 1. To address this issue, we conducted Experiment 2A, in which we manipulated WM demands by introducing an additional WM updating task into the DT situation. This allowed us to keep the number of stimulus–response mappings and, thus, the frequency of stimulus and response repetitions constant across load conditions.

## Experiment 2A

In Experiment 2A, we implemented an additional WM updating task during a DT with variable task order. In high-load blocks, participants had to maintain a number in WM and, depending on a presented arithmetical stimulus (a ‘ + ’ sign or a ‘- ‘ sign), constantly perform an arithmetical task on this number, i.e. count up or down in steps of one. In low-load blocks, we also presented these operators but participants were instructed to simply monitor the sequence of operators. Thus, in addition to the task-order set and the task sets of the component tasks, in both load conditions, participants had to maintain additional task information active in WM. However, WM demands were increased in high-load blocks relative to low-load blocks, as participants had to permanently update their WM content, i.e. the result of the ongoing arithmetical task, in high-load blocks (Soutschek et al., 2013), while there was no need to update numerical information in WM during low-load blocks. As in Experiment 1, we assumed that, if the processing of the task-order set indeed relies on WM resources, increasing WM demands should reduce the performance benefit for same-order relative to different-order trials. In addition, and in order to test if monitoring related processes do or do not rely on WM, participants also performed fixed-order blocks under both load conditions.

## Materials and methods

### Participants

Twenty-four (23 female) right-handed participants with an age range from 19 to 27 (mean age 22) from the Humboldt-Universität in Berlin and the Martin-Luther University Halle-Wittenberg took part in this experiment. Participants gave their informed consent to participate in the study at the beginning of each session. As compensation, they received either course credit or 8 euros per hour. Data of one participant were excluded due to a high number of erroneous trials (only 57% correct trials) and very poor performance in the arithmetical task (an average difference value of 4 for random order blocks, see below).

### Apparatus and stimuli

The experimental setting was similar to Experiment 1. Participants performed a DT consisting of an auditory (tone discrimination) and a visual (letter discrimination) component task. For the auditory task, one of three tones (200 Hz, 650 Hz, & 1100 Hz) was presented and participants were asked to respond to these stimuli with their left ring, middle and index finger by pressing the ‘Y’, ‘X’, and ‘C’ buttons, respectively. To not interfere with the arithmetical task, we used a letter discrimination task with the letters ‘A’, ‘E’, and ‘O’ (0.52° × 0.31°) as the visual component task. Participants were instructed to respond to these letters in ascending order by pressing the ‘,’, ‘.’, and ‘- ‘ buttons with their right index, middle, and ring finger, respectively. Analogously to Experiment 1, participants were instructed to respond to the target stimuli as fast and as accurately as possible according to the order of their presentation.

### Design and procedure

Trial timing was adjusted to account for the increased demands posed by the additional WM updating task. At the beginning of each trial, a fixation mark (either a ‘ + ’ or a ‘- ‘ sign) was presented for 1500 ms, which was then followed by both target stimuli. Each stimulus was presented for 200 ms and the onset of both stimuli was separated by a time interval of 200 ms. Trials ended after participant gave their second response or after a maximum response period of 4000 ms. The next trial started after an ITI of 1000 ms, during which feedback for erroneous and omitted responses was given.

In total, participants performed 16 blocks with random task order. As in Experiment 1, during random-order blocks, in half of the trials, the auditory stimulus was presented first while in the rest of the trials the visual stimulus was presented first. The specific order of stimuli varied randomly from trial to trial. Half of the trials were same-order and the other half different-order trials. The sequence of these same-order and different-order trials was randomized within each block. Block length was shortened in order to reduce the difficulty of the task situation constituted by the combination of the DT and the WM updating task so that each random-order block consisted of 19 trials. In sum, this resulted in 9 same-order and 9 different-order trials for each block (the additional first trial of each block was removed from analyses as it neither constitutes a same-order nor a different-order trial).

In both, low-load and high-load blocks, either a ‘ + ’ sign or a ‘- ‘ sign was presented as a fixation mark. The sequence of these operators was randomized throughout each block. In high-load blocks, participants had to keep a number in mind and constantly perform a continuous arithmetical calculation on this number based on the presented operators. Starting from the number ‘10′ at the beginning of each block, participants were instructed to either count up or down in steps of 1 in a continuous fashion. For example, if in the first trial of high-load block a ‘ + ’ sign was presented, they had to add the number ‘1′ and remember the result (’11′). If in the next trial a ‘- ‘ sign was presented, they had to retrieve the result from the previous trial (’11′) and subtract the number ‘1′ to calculate the new result (’10′). Consequently, demands on WM were increased as participants had to constantly maintain and manipulate their WM content. At the end of each block, participants were asked to give their final result of the continuous arithmetical task by writing it on a separate sheet of paper. In low-load blocks, the same arithmetical operators were presented as the fixation mark. However, participants were instructed to solely monitor the sequence of operators throughout the block without performing the additional addition/subtraction task. Consequently, demands on WM were reduced compared to high-load blocks while participants still had to perform an additional task, i.e. the monitoring of the operators. Please note, however, that since participants were not required to give any response for the monitoring task, we cannot exclude completely that participants ignored the monitoring task and only processed the DT at hand. This might be problematic because if this should be the case, low and high load block not only differ in WM load but also in the additional requirement to switch between a DT and an additional task (the WM updating task and no task in low and high load blocks, respectively). As a result, the mere switching between the applied DT and an additional task might explain any effects of our manipulation on the RT differences between same-order and different-order trials in Experiment 2A. This issue was addressed in an additional control experiment (see Experiment 2B).

During a practice phase, participants received 30 single-task trials for each component task in counterbalanced order. Single-task blocks were followed by two random-order DT blocks with 15 trials and with low demands on WM. Afterward, participants received the instructions for the high-load condition and performed three random-order blocks consisting of nine trials under high WM load. In the first part of the main experiment, participants performed 16 random-order blocks consisting of 19 trials, 8 blocks under low load and 8 blocks under high load. The sequence of low and high-load blocks was counterbalanced across participants. After random-order blocks, participants received eight fixed-order blocks with 18 trials each. Half of these blocks consisted of AUD—VIS trials, the other half of VIS—AUD trials. One half of the participants first performed these fixed-order blocks in the low-load condition and then in the high-load condition, while the remaining participants performed the low and high load fixed-order blocks in reversed order. The sequence of AUD – VIS and VIS – AUD fixed-order DT blocks was counterbalanced across participants.

## Results

Data pre-processing and analysis of DT performance were analogous to Experiment 1. Error trials (*m* = 19%), as well as trials with RTs slower and faster than ± 2.5 standard deviation from the mean of each factor combination (*m* = 2%) were removed before RT analysis for each participant. For analyzing performance in the WM updating task in high-load blocks, we calculated the difference between the correct result and the result given by participants at the end of each block.

### Working memory updating task

Participants exhibited an appropriate accuracy in the WM updating task with an average difference value of *m* = 0.77 across all blocks. Furthermore, by using a paired sample t-test, we revealed that performance in the WM updating task was impaired in random-order (average difference value of *m* = 0.96) compared with fixed-order blocks (average difference value of *m* = 0.47), *t*(22) = 3.646, *p* = 0.001, *d* = 0.763.

### Comparison between same-order and different-order trials

**Fig. 3:**
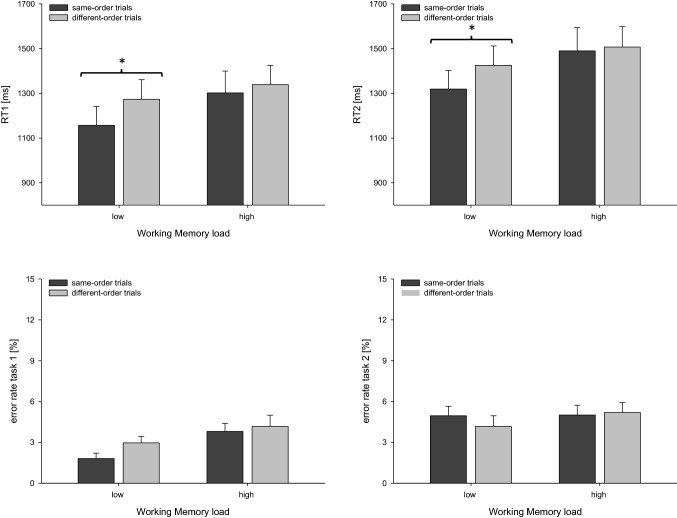
Mean RTs and error rates for task 1 and task 2 as a function of trial type and Working Memory load for Experiment 1. Error bars reflect the standard error of the mean. Asterisks indicate significant differences between same-order and different-order trials conditions. Left panels: reaction times (RT 1, upper panel) and error rates (lower panel) for task 1, right panels: reaction times (RT 2, upper panel) and error rates (lower panel) for task 2

As in Experiment 1, we conducted an ANOVA with the within-subjects factors WM LOAD (low-load blocks, high-load blocks) and TASK ORDER (same-order trials, different-order trials) on RTs and error rates. This analysis demonstrated that RT 1 was significantly longer in high-load blocks (*m* = 1320 ms) than low-load blocks (*m* = 1215 ms), *F*(1, 22) = 7.288, *p* = 0.013, *η*_*p*_^*2*^ = 0.249. Additionally, RT 1 was reduced in same-order (*m* = 1229 ms) compared to different-order trials (*m* = 1306 ms, see Fig. 3), *F*(1, 22) = 21.299, *p* < 0.001, *η*_*p*_^*2*^ = 0.492, indicating the typical finding of RT benefits for same-order versus different-order trials (De Jong, 1995).

Importantly, we replicated the results of Experiment 1. As indicated by the significant interaction of the two factors TASK ORDER and WM LOAD, *F*(1, 22) = 11.281, *p* = 0.003, *η*_*p*_^*2*^ = 0.339, the RT benefit for same-order compared to different-order trials was again modulated by the factor WM LOAD. While in low-load blocks RT 1 was significantly shorter in same-order trials (*m* = 1157 ms) compared with different-order trials (*m* = 1272 ms), *t*(22) = 6.946, *p* < 0.001, *d* = 1.452, no such benefit for same- (*m* = 1302 ms) versus different-order trials (*m* = 1339 ms) could be found in high-load blocks, *t*(22) = 1.582, *p* = 0.128, *d* = 0.328, *BF*_*01*_ = 1.545. Thus, for RT 1, high compared to low WM demands yielded a reduced performance benefit for same-order trials.

For errors in task 1, the only significant effect was found for the factor WM LOAD, *F*(1, 22) = 12.056, *p* = 0.002, *η*_*p*_^*2*^ = 0.354, indicating that errors in task 1 occurred more often in high-load (*m* = 4.0%) compared to low-load blocks (*m* = 2.5%). Neither the main effect of TASK ORDER, *F*(1, 22) = 2.063, *p* = 0.165, *η*_*p*_^*2*^ = 0.086, nor the interaction of the two factors, *F*(1, 22) = 0.692, *p* = 0.414, *η*_*p*_^*2*^ = 0.030 reached significance.

Also for RT 2, we found a significant main effect for the factor WM LOAD, *F*(1, 22) = 7.027, *p* = 0.015, *η*_*p*_^*2*^ = 0.242, with slower responses for the high-load (*m* = 1498 ms) compared to the low-load condition (*m* = 1372 ms). Additionally, we found a significant main effect for the factor TASK ORDER, *F*(1, 22) = 15.195, *p* = 0.001, *η*_*p*_^*2*^ = 0.409, indicating an RT benefit for same-order trials (*m* = 1404 ms) in contrast to different-order trials (*m* = 1466 ms).

Furthermore, the performance benefit in RT 2 for same-order relative to different-order trials differed between load conditions, *F*(1, 22) = 12.879, *p* = 0.002, *η*_*p*_^*2*^ = 0.369. The significant performance benefit for same-order trials (*m* = 1319 ms) compared to different-order trials (*m* = 1425 ms) in low-load blocks, *t*(22) = 6.632, *p* < 0.001, *d* = 1.385, could not be replicated in high-load blocks. Instead, in the latter block type, RT 2 did not differ significantly between same-order (*m* = 1490 ms) and different-order trials (*m* = 1507 ms), *t*(22) = 0.697, *p* = 0.493, *d* = 0.150, *BF*_*01*_ = 3.671.

For errors in task 2 no effect was significant, with *F*(1, 22) = 0.951, *p* = 0.340, *η*_*p*_^*2*^ = 0.041 for the factor WM LOAD, with *F*(1, 22) = 0.311, *p* = 0.583, *η*_*p*_^*2*^ = 0.014 for the factor TASK ORDER, and with *F*(1, 22) = 0.968, *p* = 0.336, *η*_*p*_^*2*^ = 0.042 for the interaction of these two factors. In sum, analyses of RTs replicated the findings of Experiment 1, consistent with our assumption that the task-order set cannot be processed efficiently in WM when WM demands are high.

### Comparison between fixed-order and random-order blocks

In addition, we separately analyzed RT 1 and RT 2 and error rates using a conventional ANOVA with the within-subjects factors WM LOAD (low-load blocks, high-load blocks) and BLOCK TYPE (fixed-order blocks, random-order blocks) and a respective Bayesian model comparison. In comparison to the low-load condition (*m* = 1050 ms), RT 1 was increased in the high-load condition (*m* = 1184 ms), *F*(1, 22) = 26.217, *p* < 0.001, *η*_*p*_^*2*^ = 0.544. Additionally, responses on task 1 were slower in random-order blocks (*m* = 1267 ms) relative to fixed-order blocks (*m* = 966 ms), *F*(1, 22) = 65.547, *p* < 0.001, *η*_*p*_^*2*^ = 0.749. Importantly, this increase from fixed-order to random-order blocks did not differ between both load conditions, as was indicated by the non-significant interaction of these two factors, *F*(1, 22) = 1.911, *p* = 0.181, *η*_*p*_^*2*^ = 0.080. This was also supported by a Bayes Factor of *BF*_*01*_ = 3.327 from the Bayesian model comparison, providing evidence for a model only containing the main effects WM LOAD and BLOCK TYPE without further specifying an interaction of these two factors.

Regarding accuracy in task 1, participant produced more errors when WM demands were high (*m* = 3.2%) compared to when they were low (*m* = 1.7%), *F*(1, 22) = 20.646, *p* < 0.001, *η*_*p*_^*2*^ = 0.484. Also, more errors could be observed in random-order (*m* = 3.2%) relative to fixed-order blocks (*m* = 1.7%), *F*(1, 22) = 37.975, *p* < 0.001, *η*_*p*_^*2*^ = 0.633. The interaction between the two factors was not significant, *F*(1, 22) = 0.283, *p* = 0.600, *η*_*p*_^*2*^ = 0.013. The non-significant interaction is also supported by a Bayes factor of *BF*_*01*_ = 3.056 providing evidence for a model that does not specify the interaction of the two factors.

For RT 2 we found similar results: RTs in high-load blocks (*m* = 1357 ms) were slower than RTs in low-load blocks (*m* = 1201 ms), *F*(1, 22) = 22.645, *p* < 0.001, *η*_*p*_^*2*^ = 0.507. Additionally, RT 2 was increased in random- (*m* = 1435 ms) compared fixed-order blocks (*m* = 1233 ms), *F*(1, 22) = 60.476, *p* < 0.001, *η*_*p*_^*2*^ = 0.733. This increase in RT 2 from fixed-order to random-order blocks did not differ between the low-load and high-load condition, as was indicated by the non-significant interaction of the two factors, *F*(1, 22) = 1.596, *p* = 0.220, *η*_*p*_^*2*^ = 0.068. Similarly, the respective model comparison yielded a Bayes factor of *BF*_*01*_ = 2.40, providing no evidence for a modulation of performance differences between both block types due to WM load.

For error rates in task 2, no significant effect of the factors WM LOAD, *F*(1, 22) = 2.649, *p* = 0.118, *η*_*p*_^*2*^ = 0.107, BLOCK TYPE, *F*(1, 22) = 1.919, *p* = 0.180, *η*_*p*_^*2*^ = 0.080, nor for their interaction, *F*(1, 22) = 0.143, *p* = 0.709, *η*_*p*_^*2*^ = 0.006. Further support for the non-significant interaction comes from the Bayesian model comparison with a Bayes Factor of, *BF*_*01*_ = 3.338. In sum, RT and error data suggest that monitoring related processes were, again, not affected by the WM manipulation. This was indicated by a lacking effect of WM load on performance decrements in random-order relative to fixed-order blocks.

In analogy to Experiment 1, we also compared RTs in the last random-order block with performance in the (succeeding) first fixed-order blocks to exclude that performance differences between both block types can be explained exclusively due to practice effects. We found that RT 1 was abruptly reduced from the last random-order block (*m* = 1282 ms) to the first fixed-order block (*m* = 979 ms), *F*(1, 22) = 17.655, *p* < 0.001, *η*_*p*_^*2*^ = 0.445. Similarly for task 2, RTs were significantly slower in the last random-order block (*m* = 1484 ms) compared with the first fixed-order block (*m* = 1145 ms), *F*(1, 22) = 6.210, *p* = 0.021, *η*_*p*_^*2*^ = 0.220. Please note, that this RT difference between the last random-order and the first fixed-order block is similar compared to contrasting random-order and fixed-order blocks collapsed over the entire experiment. Thus, these sudden improvements in performance from one block to the other do not confirm the assumption of practice effects as the only reason for performance differences between fixed-order and random-order blocks. In the next step, we compared performance in the first random-order block with performance in the last fixed-order block. Also this comparison revealed that RT 1 was slowed down in random-order (*m* = 1373 ms) compared with fixed-order blocks (*m* = 964 ms), *F*(1, 22) = 21.378, *p* < 0.001, *η*_*p*_^*2*^ = 0.493. Similarly, for RT2 a similar pattern was observed with slower RTs in random-order (*m* = 1573 ms) compared with fixed-order blocks (*m* = 1129 ms), *F*(1, 22) = 19.081, *p* < 0.001, *η*_*p*_^*2*^ = 0.464. Importantly, RT differences between the last random-order block and the first fixed-order block (task 1: *m* = 303 ms; task 2: *m* = 339 ms) did not differ compared with RT differences between the first random-order block and the last fixed-order block (task 1: *m* = 408 ms; task 2: *m* = 434 ms), *t*(22) = 0.982, *p* = 0.337, *d* = 0.201 for task 1 and *t*(22) = 0.592, *p* = 0.560, *d* = 0.123 for task 2. Additional Bayesian t-tests supported these results with *BF*_*01*_ = 2.998 for task 1 and *BF*_*01*_ = 3.901 for task 2, providing no evidence for any differences between fixed-order and random-order blocks across the course of the experiment. These findings indicate that the difference between fixed-order and random-order blocks can for the most part be accounted for by increased demands on task-order coordination.

## Discussion

In Experiment 2A, we replicated the findings of Experiment 1: When WM load was low, we found a performance benefit in task 1 and task 2 for same-order compared to different-order trials (Luria & Meiran, 2003, 2006). Increasing load due to the additional WM updating task resulted in absent performance benefits for same-order trials. Again, these results are in line with the assumption that the processing of the task-order set requires WM resources and that increasing WM load hampers the processing and maintenance of a task-order set. Importantly, in Experiment 2A, we manipulated WM demands by applying a WM updating task during DT performance. Thus, we can exclude the alternative explanation for the results of Experiment 1, according to which the decreased performance benefit for same-order trials could be accounted for by an increased number of stimulus–response repetitions in low-load compared to high-load blocks (Hommel et al., 2004; Mayr et al., 2003). Also, by observing no differences in performance decrements in random-order compared to fixed-order blocks under both load conditions, we found no evidence for the modulation of monitoring-related processes due to increased WM demands (Stelzel et al., 2008).

However, we need to address a potential alternative explanation for the results of Experiment 2A in an additional Experiment 2B. More specifically, in this experiment, we wanted to test whether switching between a DT situation and an additional task per se (i.e. irrespective of WM demands) can decrease the performance benefits for same-order relative to different-order trials. In high-load blocks of Experiment 2A, we enforced participants to perform the additional arithmetical task and prompted them to give their final result at the end of each block. In low load-blocks, in contrast, participants were instructed to simply monitor the operators and no overt response was required. Thus, there was no control regarding participants’ performance in the low-load condition. Consequently, in low-load blocks, we cannot exclude that participants might have performed the DT and avoided the additional task, i.e. monitoring the operators. This might, at least theoretically, be problematic, because then in high-load blocks, participants had to switch between a DT and a highly demanding WM updating task, whereas in low-load blocks participants may have performed only the DT without switching between the two different tasks. As a result, both conditions might not only differ in WM demands but also in the additional requirement to switch between a DT and an additional task. Importantly, this additional switching rather than increased WM may be responsible for reduced performance benefits in same-order relative to different-order trials in high-load blocks of Experiment 2A.

To elucidate whether the switching between the DT and an additional task alone can evoke the disappearance of the performance benefits for same-order trial, in Experiment 2B we incorporated a Go/NoGo task (Donders, 1969) in random-order DT blocks. We included this Go/NoGo as the additional task because it resembles the monitoring task from low-load blocks of Experiment 2A: First, the Go/NoGo task is characterized by low demands on WM. Second, to perform this task correctly, participants have to monitor the sequence of stimuli from trial to trial (and decide whether to press a button or not) which was also the core requirement for the monitoring task in low-load blocks of Experiment 2A. Furthermore, the Go/NoGo task requires an overt response (at least in some trials). Consequently, we can verify whether participants also performed the additional task or whether they only focused on the DT without performing this additional Go/NoGo task.

## Experiment 2B

The aim of Experiment 2B was to investigate, whether the switching between a random-order DT and an additional task with low WM demands alone can result in decreased performance benefits for same-order relative to different-order trials. For this purpose, we used a Go/NoGo task (Donders, 1969). Similar to the additional task in low load blocks of Experiment 2A, this Go/NoGo requires participants to monitor the sequence of stimuli while keeping demands on WM rather low. Importantly, if switching between a random-order DT and an additional Go/NoGo alone results in the disappearance of the performance benefit for same-order trials, we should find no RT difference between same-order compared with different-order trials. On the other hand, if we still find faster RTs in same-order trials despite the additional Go/NoGo task, we can conclude that switching between a DT and an additional task alone is not sufficient to reduce the performance benefit for same-order trial. Please note that the monitoring task of Experiment 2A and the Go/NoGo task of Experiment 2B might be characterized by different cognitive demands beyond WM load. In more detail, in the monitoring task participants had to monitor the sequence of stimuli while the Go/NoGo task requires participants either to execute a motor response or to inhibit this response. However, WM memory load between both tasks should be similarly low. Furthermore, we guaranteed that participants indeed perform this additional task by introducing an overt response requirement. Consequently, even if both the monitoring and Go/NoGo task do differ in their cognitive demands, we still can test whether the mere switching between two tasks is sufficient to reduce the performance difference between same-order and different-order trials (as it might have occurred in Experiment 2A).

## Materials and methods

### Participants

Twenty-four right-handed participants (17 female, mean age 22 years) from the Humboldt-Universität in Berlin, who gave their informed consent in advance, took part in Experiment 2B. As compensation, they received either course credit or 8 euros per hour.

### Design and procedure

Participants performed 12 random-order blocks from Experiment 2A. However, instead of performing an additional arithmetical or monitoring task, participants were instructed to perform a Go/NoGo task (Donders, 1969) upon the fixation mark. For this purpose, participants were asked to respond to the ‘-’-sign by pressing the space-button with their (either left or right) thumb while withholding their response in case of the ‘ + ’ sign. By applying this Go/NoGo with an overt response, we guaranteed that participants attended to the additional task while keeping WM demands to a minimum.

## Results

Analysis of the Go/NoGo task indicated appropriate performance with a mean error rate of 3.86% (SD = 5.49%) for omission errors (misses) and 0.31% (SD = 0.66%) for commission errors (false alarms). To test for a performance benefit for same-order compared to different-order trials despite the additional Go/NoGo task, RTs and error rates (separately for task 1 and task 2) were analyzed. Data preprocessing and aggregation were equivalent compared to the previous experiments. Only trials with correct responses in both tasks (*m* = 73%) were included and trials within a range of ± 2.5 standard deviation from the mean of each factor combination (*m* = 2%) were excluded from RT analyses.

### Comparison between same-order and different-order trials

To test for better performance in same-order trial versus different-order trials, we analyzed RT 1 using paired simple *t*-tests. Importantly, despite the additional Go/NoGo task, this analysis revealed faster responses in same-order (*m* = 1136 ms) compared to different-order trials (*m* = 1227 ms), t(23) = 3.272, *p* = 0.003, *d* = 0.681.^1^ Similarly, error rates in task 1 increased from same-order trials (*m* = 4.2%) to different-order trials (*m* = 6.3%), *t*(23) = 2.982, *p* = 0.007, *d* = 0.623.

A similar result was found for RT 2 with a benefit for same- (*m* = 1354 ms) compared with different-order trials (*m* = 1426 ms), *t*(23) = 2.625, *p* = 0.015, *d* = 0.553.^2^ Error rates in task 2 were not affected by a change in task order, *t*(23) = 0.909, *p* = 0.373, *d* = 0.198. Thus, RT data from task 1 and task 2 demonstrated a performance benefit for same-order relative to different-order trials also when introducing an additional Go/NoGo task with low demands on WM.

### Comparison across experiment 2A and 2B

In Experiment 2B we demonstrated faster RTs for same-order compared to different-order trials despite introducing a Go/NoGo task into a DT situation with variable task order. To further confirm this benefit for same-order trials despite a Go/NoGo task with low demands on WM, we compared RT data of Experiment 2B with the RT data from random-order blocks in the low load condition of Experiment 2A. Note that demands on WM should be similar in low-load blocks from Experiment 2A, in which participants had to monitor the operands presented at the beginning of each trial, and in Experiment 2B, in which participant had to monitor the operands *and* give a response whenever a “-” sign was presented as the fixation mark. As a result, we expected similar performance benefits for same- compared to different-order trials in both situations.

To test this assumption, we performed a frequentist ANOVA with the within-subjects factor TASK ORDER (same-order trials, different-order trials) and the between-subjects factor EXPERIMENT (Experiment 2A, Experiment 2B) on RTs and error rates in task 1 and task 2. In analogy to the previous experiments, we also performed a Bayesian ANOVA to further confirm the assumption that the performance benefit for same-order trials does not differ across experiments. For this purpose, we calculated the posterior probabilities of a model not including the interaction of the factors TASK ORDER and EXPERIMENT and compared it to a model containing this interaction. A Bayes factor BF_01_ with a value larger than 1 would provide evidence for a model not including the interaction effect indicating that the performance difference between same-order and different-order trials was similar across Experiment 2A and Experiment 2B. A Bayes factor BF_01_ with a value smaller than 1, on the other hand, would provide evidence for a model including this interaction suggesting that the performance difference between both trial types differs between both experiments.

For RTs in task 1, we found a significant effect of the factor TASK ORDER, *F*(1, 45) = 39.819, *p* < 0.001, *η*_*p*_^*2*^ = 0.469, indicating faster RT 1 in same-order (*m* = 1146 ms) compared to different-order trials (*m* = 1249 ms). Importantly, this performance benefit for same-order trials did not differ between the low-load condition of Experiment 2A and in Experiment 2B; the interaction of TASK ORDER and EXPERIMENT was non-significant, *F*(1, 45) = 0.572, *p* = 0.453, *η*_*p*_^*2*^ = 0.013. The non-significant interaction was also supported by a Bayes factor of *BF*_*01*_ = 2.989, providing evidence for a model that does not specify the interaction of the two factors. The factor EXPERIMENT did not reach significance, *F*(1, 45) = 0.099, *p* = 0.755, *η*_*p*_^*2*^ = 0.002, *BF*_*01*_ > 100. Also, when analyzing accuracy data for task 1, we could not find any evidence that the difference in error rates between same-order and different-order trials varied across Experiment 2A and Experiment 2B. This was indicated by the non-significant interaction of the factors TASK ORDER and EXPERIMENT, *F*(1, 45) = 0.977, *p* = 0.328, *η*_*p*_^*2*^ = 0.021, as well as by a Bayes factor of *BF*_*01*_ = 2.173 from the respective model comparison. Furthermore, the factor ORDER reached significance, *F*(1, 45) = 13.176, *p* = 0.001, *η*_*p*_^*2*^ = 0.226, indicating increased error rates in task 1 for different-order (*m* = 4.4%) relative to same-order trials (*m* = 2.8%) across both experiment. The effect of the factor EXPERIMENT, *F*(1, 45) = 9.042, *p* = 0.004, *η*_*p*_^*2*^ = 0.167, indicating increased error rates in task 1 in Experiment 2B (*m* = 4.9%) compared to error rates in Experiment 2A (*m* = 2.4%).

We found a similar pattern for task 2. RT 2 was significantly faster in same-order trials (*m* = 1336 ms) relative to different-order trials (*m* = 1423 ms), *F*(1, 45) = 30.809, *p* < 0.001, *η*_*p*_^*2*^ = 0.406. Importantly, this performance benefit did not differ across both experiments, as the interaction of the factors TASK ORDER and EXPERIMENT was not significant, *F*(1, 45) = 1.138, *p* = 0.292, *η*_*p*_^*2*^ = 0.025. Similarly, the respective model comparison yielded a Bayes factor of BF_01_ = 2.369, favoring a model not specifying the interaction of TASK ORDER × EXPERIMENT and providing further evidence for the assumption that performance differences between same-order and different-order trials did not differ across both experiments. Furthermore, the factor EXPERIMENT was not significant, *F*(1, 45) = 0.025, *p* = 0.874, *η*_*p*_^*2*^ = 0.001, *BF*_*01*_ > 100. For error rates in task 2, neither the factors ORDER, *F*(1, 45) = 0.013, *p* = 0.909, *η*_*p*_^*2*^ < 0.001, EXPERIMENT, *F*(1, 45) = 1.696, *p* = 0.199, *η*_*p*_^*2*^ = 0.036 nor their interaction, *F*(1, 45) = 1.661, *p* = 0.204, *η*_*p*_^*2*^ = 0.036, *BF*_*01*_ = 2.194 were significant, with the latter indicating that performance differences between same-order and different-order trials were similar in both experiments. Thus, comparing RT and error rate with data from low-load blocks of Experiment 2A further confirms that switching between an additional Go/NoGo task (with low demands on WM) and a random-order DT did not affect the performance benefits for same-order compared with different-order trials in Experiment 2B.

## Discussion

In Experiment 2B, we demonstrated that also in face of an additional Go/NoGo task with low demands on WM, performance is improved in same-order compared to different-order trials. These performance benefits did not differ from those found in the low-load condition in Experiment 2A as was confirmed by an additional comparison between both experiments and the respective Bayesian analyses. Thus, the mere demand to switch between two different tasks, i.e. the random-order DT and an additional task, does not lead to the reduction of performance benefits for same-order relative to different-order trials. Therefore, with respect to the findings of Experiment 2A, we conclude that the disappearance of the performance benefit for same-order compared to different-order trials cannot merely be explained by the need to switch between the random-order DT and the WM updating task. Instead, these results can most likely be attributed to increased WM demands in high-load blocks of Experiment 2A.

However, a potential confound of Experiment 2A that might have occurred is that participants were instructed to respond with their thumb to the Go/NoGo task. Performing this additional motor response with the thumb might have resulted in forward compatibility-like effects due to lingering motor activation after an effector repetition (e.g. response to the ‘-’-sign by pressing the space-button with the right thumb and then a first response to the visual component task of the DT situation). Importantly, however, such forward compatibility effects should mainly occur between consecutively occurring responses. Thus, this effect should mainly affect performance in the subsequent task following the Go response, i.e. task 1, but not in task 2. As we observed similar effects for task 1 and for task 2, we argue that the occurrence of these forward compatibility-like effects is rather unlikely. Furthermore, even if they had occurred, they should have had no systematic effect on the difference between same-order and different-order trials. This is so because the ‘-’-sign (indicating a go response) occurred equally often in same-order and different-order trials. As a result, same-order and different-order trials should have been affected to a similar degree by effector repetitions in go trials.

## General discussion

The aim of the present study was to investigate the role of WM for task-order coordination in DT situations. For this purpose, in the first two experiments, we introduced a WM manipulation during a DT with variable order of the component tasks. In both experiments, in low-load conditions, we found a performance benefit for same-order trials compared to different-order trials (De Jong, 1995; Luria & Meiran, 2003, 2006; Szameitat et al., 2006). In contrast, when WM load was increased, this performance benefit vanished and no difference in RTs could be observed between same-order and different-order trials. This result confirms the assumption that the processing of the task-order set relies on WM resources (Luria & Meiran, 2003, 2006; Szameitat et al., 2006). As a result, increasing WM load hampers this processing of the task order set, and the benefit for same-order versus different-order trials is reduced in high compared with low WM load conditions. In Experiment 1, this was shown by varying the number of stimulus–response mappings for each task (Stelzel et al., 2008). In Experiment 2A, we replicated the results of Experiment 1 by introducing an additional WM updating task (Soutschek et al., 2013). This was necessary, as the reduced performance benefits for same-order trials in low-load compared to high-load blocks in Experiment 1 could also be explained by different frequencies of stimulus and response repetitions (Hommel et al., 2004; Mayr et al., 2003). Furthermore, in Experiment 2B, we tested whether switching between a random-order DT and an additional task alone (rather than increased WM load) causes the disappearance of the performance benefit for same-order relative to different-order trials. Importantly, in this experiment, we still found performance benefits for same-order trials compared with different-order trials despite an additional Go/NoGo task (Donders, 1969) with low demands on WM. Thus, we conclude that the findings of Experiment 2A, can most likely not be attributed to switching between two different tasks alone but rather to increased WM demands. In sum, the results of the current study do not support the assumption that the performance benefit for same-order trials occurs due to automatic priming in long-term memory (e.g. Logan, 1988). Instead, they are consistent with the assumption that the task-order set is actively maintained and processed in WM during DT processing and, thus highlights the role of WM for task-order coordination.

In addition, we also investigated, whether WM load affects the monitoring of the stimulus sequence, which is necessary due to the instruction to respond to the tasks according to the order of stimulus presentation. To this aim, we compared the performance decrements between fixed-order blocks, in which the stimuli were presented in fixed order and monitoring was not necessary, and random-order blocks (Stelzel et al., 2008). In Experiment 1 and Experiment 2A, we demonstrated that increasing WM load did not affect monitoring related processes in DT with variable task order. This was indicated by similar RT increases from fixed-order to random-order blocks in low-load and high-load blocks and further confirmed by relevant Bayesian model comparisons.

## The role of WM for DT situations

There is ample evidence suggesting that performing more than one task simultaneously draws on WM resources, which are necessary to represent relevant task information and make this information accessible for various cognitive operations and actions (Law et al., 2013; McDowell et al., 1997; Redick et al., 2016; Todorov et al., 2018). So far, however, in the field of DT research, this view has been largely limited to the level of the component tasks. More specifically, it has been argued that specific task information of both component tasks, i.e. the task sets, have to be maintained in an active state in WM during DT processing (Huestegge & Koch, 2010; Luria & Meiran, 2006; Maquestiaux et al., 2004; Oberauer, 2009; Schubert & Strobach, 2018). The findings of the current experiments go beyond these earlier studies and add important new knowledge to the existing DT literature. More specifically, the current results indicate that not only specific information of the component tasks, i.e. the tasks sets, is maintained and processed in WM, but also higher-order information about the processing sequence of tasks. On a theoretical level, it has been argued that a task-order set, containing information of the specific task order in a given trial, is processed in WM during DTs with variable task order (Luria & Meiran, 2003, 2006; Schubert, 2008). So far, preliminary evidence for this assumption stems from the fact that RTs are faster when task order is repeated (in same-order trials) compared to when task order changes (in different-order trials) relative to the previous trials. This finding suggests that the task-order set of the preceding trial is still active in WM after it has been applied (De Jong, 1995; Kübler et al., 2018; Luria & Meiran, 2003). The findings of the current study demonstrate that the performance benefit for same-order compared to different-order trials vanishes when pushing WM capacity to its limits. This indicates that the task-order set cannot be processed efficiently in WM between two succeeding trials when the load is increased. Thus, the current results confirm, first, that sequence information about the to be processed tasks is held and processed in WM in addition to the task sets of the component tasks and, second, that factors influencing the efficiency of WM affect task order processing in DT situations.

The data of the present study are in line with current models that propose a prominent role of WM for task processing (Cowan, 1999; Oberauer, 2009, 2010; for a suggestion on the neural implementation of WM see also Brass et al., 2017). For example, the model of Oberauer (2009, 2010) conceptualizes WM as an attentional system that selects relevant task representations, e.g. task sets, and makes them accessible to guarantee goal-directed behavior. For this purpose, the model proposes that task representations sequentially pass through different levels of activation during task processing. Importantly, the higher the level of activation, the more susceptible they are to capacity limitations. In the *activated part of procedural long-term memory*, procedural representations are activated at a subthreshold level. This component of WM has a rather large capacity, however, representations cannot gain direct control over cognitive operations or actions. For this purpose, representations have to enter the second level of activation, the *bridge*. The *bridge* holds task information and task sets that are “currently in control of thought and action” (Oberauer, 2009, p. 58) and makes them directly accessible for operation in the third level, the *response focus*. Increased activation in and access to implementation in the response focus, however, go along with a limited capacity of the bridge. As a result, only a restricted amount of information can be maintained at this level. Consequently, the amount of task information which can be maintained active in the bridge depends on the load it imposes on WM with simple tasks allowing all information to be transferred concurrently into the bridge, whereas with increasing load only partial task information can be maintained (Brass et al., 2017). In addition to these storage mechanisms, Oberauer (2009) further proposes executive processes that regulate the content of WM and protect it against interference from task-irrelevant information. To do so, these executive processes can manipulate the activation levels of relevant task representations or update them in the bridge as a response to changes in the task environment.

Importantly, the findings of Experiment 1 and Experiment 2A are in line with the proposed storage and executive mechanisms underlying WM as proposed by Oberauer (2009). In more detail, in low-load conditions of the current experiments, the task-order set can be maintained in the bridge together with other task-relevant information, such as the task sets of the component tasks. As a result, in same-order trials, the task-order set of the previous trial, which still resides in the bridge, can be easily re-applied and uploaded in the response focus so that no or only a little additional executive processes are necessary. In different-order trials, however, since the task-order set of the previous trial does not specify the correct order on the current trial, additional executive mechanisms need to be employed to update or activate a new task-order set in the bridge resulting in slowed RTs compared to same-order trials. In contrast, when the load was high, either due to the increased number of stimulus–response mappings (Experiment 1) or additional task demands (Experiment 2A), it seems that the task-order set is not maintained in the bridge during the entire course of a DT trial. As a result, also in same-order trials, the task-order set of the previous trial cannot be re-applied and instead additional executive processes are necessary for implementing a new order set into the bridge. Consequently, under the high load condition, processing demands are similar in same-order and different-order trials, with both trial types requiring the employment of executive control processes for instantiating a task-order set in the bridge. This, in turn, resulted in absent performance benefits compared with different-order trials in the current study. In sum, based on the results of this study, we can conclude that not only specific task information but also information about the sequence or order of several tasks is processed and maintained in WM during DTs.

Overall, the findings of this study provide additional evidence for the assumption that task order in DT situations is actively regulated by additional executive control processes. Classical response selection models assume that the processing sequence of tasks is simply determined by which task processing stream finishes perceptual processing first and arrives at the bottleneck first. In addition, and consistent with the other DT literature, we argue that bottleneck processing does not only result from a passive occupation of the response selection stage by one of the two task processing streams. Instead, the bottleneck processing results from the occurrence of additional task-order coordination processes that rely on the explicit order information, i.e. the task order set, and that regulate task order in a top-down manner (De Jong, 1995; Luria & Meiran, 2003; Sigman & Dehaene, 2006; Szameitat et al., 2002). This account is also supported by computational models of cognitive control in DT situations that implement different control parameters for different stages of task processing, such as stimulus identification, response selection, but also planning the sequence of multiple component tasks (Logan & Gordon, 2001; Meiran et al., 2008; Meyer & Kieras, 1997).

It is important to note that these task-order coordination processes are not only employed for the special case of a DT with random stimulus order (as it was applied in the current study). Instead, it seems that these processes are also employed in DTs with constant order of tasks, such as the psychological refractory period paradigm (De Jong, 1995; Schubert, 2008). Evidence for this assumption stems, for example, from neuroimaging studies. In their study, Szameitat et al. (2002; see also Schubert & Szameitat, 2003) compared neural activation during single-task situations with neural activation during fixed-order as well as random-order DT situations. They found increased activation in the lateral prefrontal cortex (lPFC), a brain region associated with executive control processes (Brass et al., 2005; Derrfuss et al., 2004), in fixed-order and random-order blocks, but there was no such activation in single-task blocks. According to the authors, this result suggests that both DTs with fixed and DT with random task order require the employment of the same executive processes that regulate the order of task processing. Thus, the requirement for active task-order coordination processes seems to be a general characteristic of DT situations. The findings of this study add important new insights on these task-order coordination processes in DT situations, namely that these processes rely on WM resources.

However, an alternative explanation for our findings requires further consideration. In more detail, rather than being stored and actively processed in WM, it might be possible that WM is involved in binding processes as suggested by Hommel (2004; see also Frings et al., 2020). This would mean that task-order information of the prior task experience is automatically stored in temporary short-term bindings (rather than in WM) but that WM resources are necessary for the instantiation or the retrieval of these bindings. Importantly, this account predicts the same pattern of results compared to our WM hypothesis: under high compared to low WM load temporary bindings containing order information cannot be instantiated or retrieved resulting in a reduction of performance benefits for same-order compared with different-order trials. Although theoretically possible, we argue that this explanation is rather unlikely for the case of the present study. First, to our knowledge, there is no empirical evidence supporting the assumption that the instantiation or retrieval of temporary bindings rely on WM processes. Second, from a recent study, there is empirical evidence that short-term bindings do not include information about the temporal order or sequence of events (Möller & Frings, 2019). In this study, the authors could show that binding effects can also be observed if the responses to two probes are reversed compared to the responses to two primes. Based on this result, they argued that short-term bindings do not include temporal order information and that this information must be stored elsewhere. In fact, our data suggests that this order information is stored and actively processed in WM.

### Monitoring related processes and WM

While increasing WM load does affect the storage and processing of the task-order set, the current WM manipulation did not affect monitoring related processes that are required to perform both tasks according to the order of stimulus presentation. This is in line with results from a study by Stelzel et al. (2008) in which the authors employed functional magnetic resonance imaging. In this study, the authors compared fixed-order and random-order blocks under low and high WM load by using a similar manipulation as we did in Experiment 1. As a result, they demonstrated that monitoring related processes, mirrored by contrasting fixed-order and random-order blocks, and WM related processes, reflected by contrasting low-load and high-load blocks, rely on dissociable brain structures. More specifically, increasing WM load was associated with increased brain activation in caudal parts of the premotor cortex and the anterior insula. Monitoring, on the other hand, was correlated with increased activation in more anterior parts of the prefrontal cortex surrounding the inferior frontal sulcus. According to Stelzel et al. (2008), this result pattern suggests that monitoring and maintenance of task-order information are dissociable processes that are implemented by different brain structures.

Further support for the fact that monitoring and task-order set processing are indeed distinct mechanisms comes from a study of Kübler et al. (2018). In this study, the authors applied a random-order DT and varied the task-order instruction participants had to adhere to during DT processing. While in one condition participants were instructed to respond to both tasks according to the order of stimulus presentation, in the other they could freely decide in which order to perform the tasks. As a result, in the former condition participants had to employ monitoring related processes, to adjust their processing order to the stimulus sequence, whereas in the latter condition there was no need to monitor the order of stimuli presentation. As a result, the performance difference between fixed-order and random-order blocks was reduced when participants could freely decide about task order compared with when they had to match task order to the stimulus sequence. Kübler et al. (2018) concluded this result indicates that changing the instruction in DT situations can affect monitoring-related processes. In contrast, the difference between same-order and different-order trials, which reflects the processing of the task-order set in WM, did not differ between both conditions and, thus, was not affected by the instruction manipulation. According to the authors, this dissociation indicates that the performance difference between fixed-order and random-order blocks on the one, and the performance difference between same-order and different-order trials on the other hand might reflect independent mechanisms of task-order coordination, i.e. the monitoring related processes and the processing of the task-order set in WM. The results of the present study confirm the assumptions of these previous studies. More specifically, by demonstrating in two experiments that varying WM load does affect the efficient employment and processing of the task-order set in WM but not the monitoring of the stimulus sequence, we provide further evidence for the fact that monitoring and processing of order information in WM are dissociable processes that both are necessary for scheduling the sequence of task processing in DT situations.

## Conclusion

In the present study, we investigated the role of WM for task-order coordination in DT situations. For this purpose, in a series of experiments, we varied WM load during DT blocks with variable task-order. We demonstrated that increasing WM load results in reduced performance benefits for same-order trials relative to different-order trials. This confirms our assumption that task-order information cannot be maintained in an accessible state when WM capacity is at its limits and, thus, highlights the role of WM for task-order coordination.

## Data Availability

The material used and the datasets generated and analyzed during the current study are available from the corresponding author on request.

## Footnotes

1 A supplementary ANOVA with the factor TASK ORDER (same-order trial, different-order trial) and the additional factor Go/NoGo (go trials, no go trials) demonstrated that the benefit for same-order compared to different-order trials did not differ between go-trials and no-go trials in task 1. This was indicated by the non-significant effect of the interaction between TASK ORDER and Go/NoGo, *F*(1, 23) = 1.304, *p* = .265, *η*_*p*_^*2*^ = .054. Similarly, a Bayesian analysis was in favor of a model not including this interaction compared to a model specifying it as was indicated by a Bayes factor of *BF*_*01*_ = 2.861. The main effects of TASK ORDER, *F*(1, 23) = 10.706, *p* = .003, *η*_*p*_^*2*^ = .318, and Go/NoGo, *F*(1, 23) = 4.26, *p* = .051, *η*_*p*_^*2*^ = .16, reached significance, indicating faster RT1 in same-order and no go trials, respectively.

2 A supplementary ANOVA with the factor TASK ORDER (same-order trial, different-order trial) and the additional factor Go/NoGo (go trials, no go trials) demonstrated that the benefit for same-order compared to different-order trials was equal for go-trials and no-go trials in task 2. This was indicated by the non-significant effect of the interaction between TASK ORDER and Go/NoGo, *F*(1, 23) = .696, *p* = .413, *η*_*p*_^*2*^ = .029. Similarly, a Bayesian analysis was in favor of a model not including this interaction compared to a model specifying it as was indicated by a Bayes factor of *BF*_*01*_ = 3.041. Furthermore, the main effects of TASK ORDER, *F*(1, 23) = 6.892, *p* = .015, *η*_*p*_^*2*^ = .231, and Go/NoGo, *F*(1, 23) = 5.760, *p* = .025, *η*_*p*_^*2*^ = .200, reached significance, indicating faster RT2 in same-order and no go trials, respectively.

## References

[CR1] Baddeley A (2003). Working memory: Looking back and looking forward. Nature Reviews Neuroscience.

[CR2] Brass M, Derrfuss J, Forstmann B, von Cramon DY (2005). The role of the inferior frontal junction area in cognitive control. Trends in Cognitive Sciences.

[CR3] Brass M, Liefooghe B, Braem S, De Houwer J (2017). Following new task instructions: Evidence for a dissociation between knowing and doing. Neuroscience and Biobehavioral Reviews.

[CR4] Cowan N, Miyake A, Shah P (1999). An embedded-processes model of working memory. Models of Working Memory: Mechanisms of Active Maintenance and Executive Control.

[CR5] Cowan N (2010). The magical mystery four: How is working memory capacity limited, and why?. Current Directions in Psychological Science.

[CR6] De Jong R (1995). The role of preparation in overlapping-task performance. Quarterly Journal of Experimental Psychology. A, Human Experimental Psychology.

[CR7] Derrfuss J, Brass M, von Cramon YD (2004). Cognitive control in the posterior frontolateral cortex: Evidence from common activations in task coordination, interference control, and working memory. NeuroImage.

[CR8] Donders FC (1969). On the speed of mental processes. Acta Pathologica, Microbiologica, et Immunologica Scandinavica.

[CR9] Ellenbogen R, Meiran N (2008). Working memory involvement in dual-task performance: Evidence from the backward compatibility effect. Memory & Cognition.

[CR10] Fischer R, Plessow F (2015). Efficient multitasking: Parallel versus serial processing of multiple tasks. Frontiers in Psychology.

[CR11] Hick WE (1952). On the rate of gain of information. The Quarterly Journal of Experimental Psychology.

[CR12] Hirsch P, Nolden S, Koch I (2017). Higher-order cognitive control in dual tasks: Evidence from task-pair switching. Journal of Experimental Psychology: Human Perception and Performance.

[CR13] Hirsch P, Nolden S, Philipp AM, Koch I (2018). Hierarchical task organization in dual tasks: Evidence for higher level task representations. Psychological Research Psychologische Forschung.

[CR14] Hommel B (1998). Automatic stimulus-response translation in dual-task performance. Journal of Experimental Psychology: Human Perception and Performance.

[CR15] Hommel B (2004). Event files: Feature binding in and across perception and action. Trends in Cognitive Sciences.

[CR16] Hommel B, Eglau B (2002). Control of stimulus-response translation in dual-task performance. Psychological Research Psychologische Forschung.

[CR17] Hommel B, Proctor RW, Vu K-PL (2004). A feature-integration account of sequential effects in the Simon task. Psychological Research Psychologische Forschung.

[CR18] Huestegge L, Koch I (2010). Crossmodal action selection: evidence from dual-task compatibility. Memory & Cognition.

[CR19] Kikumoto A, Mayr U (2017). The nature of task set representations in working memory. Journal of Cognitive Neuroscience.

[CR20] Koch I, Poljac E, Müller H, Kiesel A (2018). Cognitive structure, flexibility, and plasticity in human multitasking—An integrative review of dual-task and task-switching research. Psychological Bulletin.

[CR21] Kübler S, Reimer CB, Strobach T, Schubert T (2018). The impact of free-order and sequential-order instructions on task-order regulation in dual tasks. Psychological Research Psychologische Forschung.

[CR22] Kübler S, Soutschek A, Schubert T (2019). The causal role of the lateral prefrontal cortex for task-order coordination in dual-task situations: A Study with Transcranial Magnetic Stimulation. Journal of Cognitive Neuroscience.

[CR23] Law AS, Trawley SL, Brown LA, Stephens AN, Logie RH (2013). The impact of working memory load on task execution and online plan adjustment during multitasking in a virtual environment. The Quarterly Journal of Experimental Psychology.

[CR24] Leonhard T, Fernandez SR, Ulrich R, Miller J (2011). Dual-task processing when task 1 is hard and task 2 is easy: Reversed central processing order?. Journal of Experimental Psychology: Human Perception and Performance.

[CR25] Logan GD (1988). Toward an instance theory of automatization. Psychological Review.

[CR26] Logan GD (2002). An instance theory of attention and memory. Psychological Review.

[CR27] Logan GD, Gordon RD (2001). Executive control of visual attention in dual-task situations. Psychological Review.

[CR28] Luria R, Meiran N (2003). Online order control in the psychological refractory period paradigm. Journal of Experimental Psychology: Human Perception and Performance.

[CR29] Luria R, Meiran N (2006). Dual route for subtask order control: Evidence from the psychological refractory paradigm. The Quarterly Journal of Experimental Psychology.

[CR30] Maquestiaux F, Hartley AA, Bertsch J (2004). Can practice overcome age-related differences in the psychological refractory period effect?. Psychology and Aging.

[CR31] Maquestiaux F, Laguë-Beauvais M, Bherer L, Ruthruff E (2008). Bypassing the central bottleneck after single-task practice in the psychological refractory period paradigm: Evidence for task automatization and greedy resource recruitment. Memory & Cognition.

[CR32] Mayr U, Awh E, Laurey P (2003). Conflict adaptation effects in the absence of executive control. Nature Neuroscience.

[CR33] Mayr U, Bryck RL (2005). Sticky rules: Integration between abstract rules and specific actions. Journal of Experimental Psychology: Learning, Memory, and Cognition.

[CR34] McCann RS, Johnston JC (1992). Locus of the single-channel bottleneck in dual-task interference. Journal of Experimental Psychology: Human Perception and Performance.

[CR35] McDowell S, Whyte J, D'Esposito M (1997). Working memory impairments in traumatic brain injury: Evidence from a dual-task paradigm. Neuropsychologia.

[CR36] Meiran N, Kessler Y, Adi-Japha E (2008). Control by action representation and input selection (CARIS): A theoretical framework for task switching. Psychological Research Psychologische Forschung.

[CR37] Meyer DE, Kieras DE (1997). A computational theory of executive cognitive processes and multiple-task performance: Part 2. Accounts of psychological refractory-period phenomena. Psychological Review.

[CR38] Möller B, Frings C (2019). Lost time: Bindings do not represent temporal order information. Psychonomic Bulletin & Review.

[CR39] Navon D, Miller J (2002). Queuing or sharing? A critical evaluation of the single-bottleneck notion. Cognitive Psychology.

[CR40] Oberauer K (2009). Chapter 2 Design for a working memory. Psychology of Learning and Motivation.

[CR41] Oberauer K (2010). Declarative and procedural working memory: Common principles, common capacity limits?. Psychologica Belgica.

[CR42] Oberauer K, Souza AS, Druey MD, Gade M (2013). Analogous mechanisms of selection and updating in declarative and procedural working memory: Experiments and a computational model. Cognitive Psychology.

[CR43] Pashler H (1994). Dual-task interference in simple tasks: data and theory. Psychological Bulletin.

[CR44] Pashler H, Johnston JC (1989). Chronometric evidence for central postponement in temporally overlapping tasks. The Quarterly Journal of Experimental Psychology.

[CR45] Raftery AE (1995). Bayesian model selection in social research. Sociological methodology.

[CR46] Redick TS, Shipstead Z, Meier ME, Montroy JJ, Hicks KL, Unsworth N, Engle RW (2016). Cognitive predictors of a common multitasking ability: Contributions from working memory, attention control, and fluid intelligence. Journal of Experimental Psychology: General.

[CR47] Schneider DW, Logan GD (2005). Modeling task switching without switching tasks: A short-term priming account of explicitly cued performance. Journal of Experimental Psychology: General.

[CR48] Schubert T (2008). The central attentional limitation and executive control. Frontiers in Bioscience.

[CR49] Schubert T, Strobach T (2018). Practice-related optimization of dual-task performance: Efficient task instantiation during overlapping task processing. Journal of Experimental Psychology: Human Perception and Performance.

[CR50] Schubert T, Szameitat AJ (2003). Functional neuroanatomy of interference in overlapping dual tasks: An fMRI study. Brain Research Cognitive Brain Research.

[CR51] Sigman M, Dehaene S (2006). Dynamics of the central bottleneck: Dual-task and task uncertainty. PLoS Biology.

[CR52] Soutschek A, Strobach T, Schubert T (2013). Working memory demands modulate cognitive control in the Stroop paradigm. Psychological Research Psychologische Forschung.

[CR53] Stelzel C, Kraft A, Brandt SA, Schubert T (2008). Dissociable neural effects of task order control and task set maintenance during dual-task processing. Journal of Cognitive Neuroscience.

[CR54] Strobach T, Hendrich E, Kübler S, Müller H, Schubert T (2018). Processing order in dual-task situations: The “first-come, first-served” principle and the impact of task order instructions. Attention, Perception, & Psychophysics.

[CR55] Strobach T, Kübler S, Schubert T (2019). Endogenous control of task-order preparation in variable dual tasks. Psychological Research Psychologische Forschung.

[CR56] Strobach T, Soutschek A, Antonenko D, Floel A, Schubert T (2015). Modulation of executive control in dual tasks with transcranial direct current stimulation (tDCS). Neuropsychologia.

[CR57] Szameitat AJ, Lepsien J, von Cramon DY, Sterr A, Schubert T (2006). Task-order coordination in dual-task performance and the lateral prefrontal cortex: An event-related fMRI study. Psychological Research Psychologische Forschung.

[CR58] Szameitat AJ, Schubert T, Muller K, Von Cramon DY (2002). Localization of executive functions in dual-task performance with fMRI. Journal of Cognitive Neuroscience.

[CR59] Todorov I, Kubik V, Carelli MG, Del Missier F, Mäntylä T (2018). Spatial offloading in multiple task monitoring. Journal of Cognitive Psychology.

[CR60] Töllner T, Strobach T, Schubert T, Müller H (2012). The effect of task order predictability in audio-visual dual task performance: Just a central capacity limitation?. Frontiers in Integrative Neuroscience.

[CR61] Tombu M, Jolicoeur P (2003). A central capacity sharing model of dual-task performance. Journal of Experimental Psychology: Human Perception and Performance.

[CR62] van den Bergh D, Van Doorn J, Marsman M, Draws T, Van Kesteren E-J, Derks K, Gupta KN (2020). A tutorial on conducting and interpreting a Bayesian ANOVA in JASP. LAnnee Psychologique.

[CR63] Wagenmakers EJ (2007). A practical solution to the pervasive problems of p values. Psychonomic Bulletin & Review.

[CR64] Wagenmakers EJ, Love J, Marsman M, Jamil T, Ly A, Verhagen J, Boutin B (2018). Bayesian inference for psychology Part II: Example applications with JASP. Psychonomic Bulletin & Review.

[CR65] Waszak F, Hommel B, Allport A (2003). Task-switching and long-term priming: Role of episodic stimulus–task bindings in task-shift costs. Cognitive Psychology.

[CR66] Welford AT (1952). The ‘psychological refractory period’and the timing of high-speed performance—a review and a theory. British Journal of Psychology. General Section.

